# Blood Pressure Control and Antihypertensive Treatment among Hemodialysis Patients—Retrospective Single Center Experience

**DOI:** 10.3390/medicina57060590

**Published:** 2021-06-08

**Authors:** Piotr Skonieczny, Zbigniew Heleniak, Marek Karowiec, Stanisław Zajączkowski, Leszek Tylicki, Alicja Dębska-Ślizień, Przemysław Rutkowski

**Affiliations:** 1 Department of Nephrology, Transplantology and Internal Medicine, Medical University of Gdańsk, 80-210 Gdańsk, Poland; zbigniew.heleniak@gumed.edu.pl (Z.H.); marek.karowiec@gumed.edu.pl (M.K.); leszek.tylicki@gumed.edu.pl (L.T.); alicja.debska-slizien@gumed.edu.pl (A.D.-Ś.); 2 Department of Physiology, Medical University of Gdańsk, 80-210 Gdańsk, Poland; stanislaw.zajaczkowski@gumed.edu.pl; 3 Department of Internal and Pediatric Nursing, Medical University of Gdańsk, 80-210 Gdańsk, Poland; przemyslaw.rutkowski@gumed.edu.pl; 4 Diaverum Dialysis Center, 80-104 Gdańsk, Poland

**Keywords:** hemodialysis, hypertension, ß-blocker, diuretic, angiotensin-converting enzyme inhibitor, renin–angiotensin–aldosterone blockade

## Abstract

*Background and Objectives*: Hypertension affects at least 80% of hemodialysis patients. Inappropriate control of blood pressure is mentioned as one of the essential cardiovascular risk factors associated with development of cardiovascular events in dialysis populations. The aim of the cross-sectional, retrospective study was the evaluation of the antihypertensive treatment schedule and control of blood pressure in relation to the guidelines in the group of hemodialysis patients. Additionally, we assessed the level of decrease in blood pressure by each group of hypotensive agents. *Materials and Methods*: 222 patients hemodialyzed in a single Dialysis Unit in three distinct periods of time—2006, 2011, and 2016—with a diagnosis of hypertension were enrolled in the study. The analysis of the antihypertensive treatment was based on the medical files and it consisted of a comparison of the mean blood pressure results reported during the six consecutive hemodialysis sessions. *Results*: The mean values of blood pressure before hemodialysis were as follows: 134/77, 130/74, and 140/76 mmHg, after hemodialysis 124/74, 126/73, and 139/77 mmHg in 2006, 2011, and 2016 respectively. The goal of predialysis blood pressure control (<140/90) was achieved by up to 64.3% of participants in 2006 as compared to 49.4% in 2016. Additionally, the postdialysis goal (<130/90) reached 57.1% of the study population in 2006 as compared to 27.1% of patients in 2016. The differences in percentage of patients using single, double, triple, and multidrug therapy during observation were not statistically significant. The most often used drugs were ß-blockers, diuretics, and calcium channel blockers in all points of the study. Blockades of the renin–angiotensin–aldosterone system in 2006 and calcium channel blockers in 2011 and 2016 were the drugs with highest impact on lowering blood pressure. *Conclusions*: The goal of predialysis or postdialysis blood pressure control was achieved in a lower percentage of patients during the period of the study. Blockade of renin–angiotensin–aldosterone system and calcium channel blockers decrease the blood pressure significantly. It is necessary to achieve better control of blood pressure in prevention of cardiovascular incidents.

## 1. Introduction

Hypertension (HT) is one of the major health problems of today’s world. Among the hemodialysis patients (HDp), HT is also one of the most common comorbidities. According to the literature, it affects at least 80% of the HDp population [1,2,3]. On the one hand, HT may be caused by chronic kidney disease (CKD). However, on the other hand, HT can also lead to CKD and even to end-stage renal disease (ESRD) and the necessity of renal replacement therapy. Hypertensive nephropathy is one of the main causes of ESRD [4]. Pathogenesis of HT is multifactorial amongst the patients with CKD because of the presence of additional risk factors such as retention of water and salt, activation of the sympathetic nerve system, and renin–angiotensin–aldosterone system (RAAS) [5,6]. Therefore, we need a multidrug therapy and individual approach to the patient to achieve proper blood pressure (BP) control [2]. Inappropriate control of HT is mentioned as one of the essential cardiovascular risk factors associated with development of cardiovascular events in the general population and dialysis patients [7]. It was found that, among patients with ESRD, the rise in mean BP of each 10mmHg is an independent cause of the development of ischemic heart disease and cardiac failure [8].

The pharmacological treatment of HT is based on five main groups of medicines—β-blockers (BB), calcium channel blockers (CCB), RAAS blockers, such as angiotensin-converting enzyme inhibitors (ACEI) and angiotensin-receptor blockers (ARBs) called also sartans (AGTR1 blocker) and diuretics. All of them are used in the treatment of hypertensive HDp [9]. Moreover, the achievement of goals of HT therapy is quite often associated with polytherapy. There are not any special guidelines concerning hypertensive treatment in HDp [5]. The National Kidney Foundation Kidney Disease Outcomes and Quality Initiative (NKF KDOQI) in guidelines from 2005 recommends the blood pressure (BP) value should be as follows: <140/90 mmHg in predialysis and <130/80 mmHg postdialysis [10]. According to the ESH/ESC 2013 guidelines, which were current for the moment of the study, the goal of treatment of systolic blood pressure (SBP) in patients with CKD should be < 140 mmHg with lowering to <130 mmHg in case of proteinuria [11]. The newest guidelines recommends a target BP  < 130/80 mmHg regardless of stage of CKD [12].

The aim of the cross-sectional, retrospective study was to evaluate the antihypertensive treatment schedule and the control of BP in relation to the guidelines in the group of HDp in the years 2006, 2011, and 2016. Additionally, we assessed the blood pressure-lowering effect of each group of drugs. 

## 2. Materials and Methods

222 patients were enrolled to the study group of HDp in a single Dialysis Unit placed in the Pomeranian District of Poland. The retrospective analysis of the antihypertensive treatment was based on the medical files and it comprises the comparison of the mean results of BP reported in six consecutive hemodialysis. BP was measured before needles placing in fistula or connecting lines to the catheter after a minimum 5 min of rest before the session. Postdialysis BP was measured after removing of needles or disconnecting lines from the catheter. No drugs were taken by the patients during the dialysis. Moreover, sex, age, diabetic, and cardiovascular status, and current treatment were included to analysis.

HT was diagnosed when systolic blood pressure (SBP) was ≥ 140 mmHg or diastolic blood pressure (DBP) was ≥ 90 mmHg, or antihypertensive drugs were prescribed. Patients without HT and patients hemodialyzed as a result of acute kidney injury were excluded from the study.

### Statistical Analyses

Data were evaluated using a GraphPad Prism (version 7) software package. In order to measure the differences, we used the U Mann Whitney test and Kruskal–Wallis with Dunn post-hoc test. In order to measure differences in BP before and after hemodialysis we used the Wilcoxon test. A *p* < 0.05 was considered statistically significant. The influence of drugs on blood pressure was evaluated by linear regression.

## 3. Results

The years 2006, 2011, and 2016 included 56, 81, and 85 HDp in the analysis, respectively. The general characteristics of the studied groups are presented in Table 1. The differences in the average age were statistically significant (61.4 in 2006, 64.4 in 2011 and 66.2 years in 2016). The prevalence of both diabetes and cardiovascular diseases (CV) had a downward tendency, but the results were not statistically significant between patients in assessed years (Table 1).

It is worth underlining that the period of time of hemodialysis was significantly longer in HDp assessed in 2016 and (45 months) as compared to 2006 (23.4 months) and 2011 (33.7 months). Furthermore, the dialysis adequacy ratio (Kt/V) improved significantly during this period of time. Also, there was no significant difference between vascular access and giving of erythropoietin (Table 1). In our study, we observed the highest value of pre- and postdialysis SBP was in 2016. The values of DBP did not differentiate between groups of patients in the period of time (Table 2). 

BP ≤ 140/90 mmHg as the goal of predialysis BP was reached more often in 2006 (64.3%), but the difference was not statistically significant as compared to 2011 and 2016 (64.2%, 49.4% respectively). Moreover, BP ≤ 130/80 mmHg as the goal for postdialysis BP, was reached statistically significant more often in 2006 (Table 2).

The average number of antihypertensive agents taken by patients increased from 2.25 to 2.53 in evaluated periods of time 2006 to 2016, respectively (Table 3).

Monotherapy was used less often in 2016 (14.1%) as a compare to 2006 (23.2%), usage of 2 and 3 hypertensive drugs were the most common in 2011 (34.6%) and in 2016 (30.6%) respectively, but there were not significant differences between the groups (Table 3).

BB were the most commonly used drugs in all periods of time (Table 4). In the study population in the second and third survey the increase of the usage of diuretics was observed. CCB and alpha-blockers administration increased slightly, but the difference was not statistically significant.

On the other hand, there was a significant decrease in the administration of ACEI from 39.3% in 2006 to 20% in 2016. However, the ARBs were administered in 2011 and 2016, therefore the overall usage of RAAS blockade was stable. RAAS blockade was applied in one third of the study population.

The linear regression analysis showed the most significant lowering BP by RAAS blockers in 2006 and CCB in 2011 and 2016 (Table 5). In the study population who achieved targets of blood pressure control in the year 2006 and 2016, CCBs were used in almost 50%. CCB in the year 2011 and RAAS blockade during the whole observation were used by 30% of patients with appropriate control of blood pressure.

## 4. Discussion

In our study, the goal of BP control according to recommendations was achieved only in half of the patients, especially in 2016, despite the fact that usage of antihypertensive drugs increased. RAAS blockers and calcium-channel blockers decreased the blood pressure significantly as compared to others hypertensive agents.

According to the review performed by Agarwal et al. in 2003 among 2535 clinically stable adult HDp with routine BP measurements prevalence of HT was 86% and only 30% of hypertensive patients have well controlled BP [13]. In the study performed by the same author in 2011 using ambulatory BP measurements among 369 chronic HDp prevalence of hypertension was 89% and was adequately controlled only in 38% [14]. In our study, the control of BP was not satisfactory in almost 50% of participants. However, this was a better result than in the review cited above. 

In the last decades, the recommendation on the treatment of HT were still based on physicians/hemodialysis unit, individual efficacy, tolerability and medical comorbidity due to the lack of randomized controlled trials. Clinicians who are taking care of HDp face the fact that, despite additional risk factors, this group of patients do not have special guidelines based on randomized studies. As a result, most doctors rely on guidelines that are prepared for the general population and extrapolated the goal of BP to HDp. A report from 2009 suggests that there is a ‘U-shaped’ association between prehemodialysis BP and mortality [15]. However, specific goals of BP for HDp are not mentioned. It is worth underlining that all studies cited in our article had been performed before ESH/ESC 2018 guidelines were prepared.

Additionally, other important consequences of inappropriate BP control are higher CV morbidity and mortality [2]. Therefore, the scientists have been looking for the parameters (i.e., the level of interleukin 6), which may improve the prediction of CV mortality in hypertensive populations [16].

### 4.1. Modality of Hypertensive Medication

In our study, the majority of HDp (62.5% in 2006, 66.7% in 2011, 74.1% in 2016) were treated by BB followed by CCB. According to meta-analyses, BBs are an effective therapeutic strategy compared with placebo because they reduce the risk of major CV events [17]. They are especially indicated for treatment of post-myocardial infarction patients suffering from symptomatic angina, heart failure with reduced ejection fraction, and for heart rate control. According to the fact that HDp have increased CV risk and CV diseases occur more often in the older age, such common BB administration in the studied population is not something unexpected. In the cited above Agarwal et al. study showed that in the United States BBs are the third most common drug for hypertension in HDp with number of 39% [13]. Previous studies suggest that BBs should be used as the first-line therapy in dialysis patients according to increased sympathetic activity [18,19]. In our study, the analysis showed a statistically significant increase in the percentage of patients who received diuretics in subsequent years. These increases can be explained as a method of treatment of HT because of higher values of BP and worse control of HT treatment in the subsequent years. Notwithstanding, the usage of diuretics in almost 70% in HDp in 2016 is unaccountable. Although we suggest that these drugs were used in accordance with the presence of residual renal function (RRF), but in our study, RRF was not measured. DOPPS study showed that worldwide use of diuretic decreased just after start of dialysis therapy [20]. The analysis in this study showed that diuretics are associated with better control of level of potassium, lower interdialytic weight gain, and lower mortality risk. In conclusion, usage of diuretics by HDp who still have RRF is beneficial for the patients and should be continued. However, we think that, in our study, diuretics were still prescribed as a continuation of hypertensive treatment that was administered before starting hemodialysis, despite the RRF being absent. Use of diuretics among anuric HDp is generally ineffective [21,22] and can cause serious side effects, i.e., ototoxicity [23]. RAAS blockade was applied in approximately one third of HDp but there was a change in the used drugs between 2006 and 2016. Agarwal showed that add-on losartan did not improve values of BP [24]. In our study, the analysis shows the best drugs for lowering blood pressure are RAAS blockers and CCB. According to ESH/ESC 2018 guidelines, the RAAS blockade are recommended as first-line therapy for CKD patients together with a CCB or diuretic [12]. Guidelines do not mark out the HDp group. However, in our study, RAAS blockers were placed at fourth place amongst used drugs. It was caused probably by the doctors afraid of hyperkalemia and therapy used before start of hemodialysis. On the other hand, CCB were administered in almost 50% of the study population. In the above-mentioned study performed by Agarwal, RAAS blockers were used in 46% of patients and were the second most common group in HT treatment [13]. RAAS blockers have proven beneficial effect on CV diseases in general population [25]. Definitely, further studies are needed to check advantages and side effects of RAAS blockers among HDp. Also in the Unites States CCB were the most often used antihypertensive medications used by 61% of patients [13].

### 4.2. Blood Pressure Control 

In our study, the average number of drugs increased from 2.25 in 2006 to 2.53 in 2016. Among patients with CKD in large clinical studies average 3.5 drugs were necessary to optimize value of BP [26]. However, Wyskida et al. revealed that the average number of hypotensive drugs was 2.1 in HDp population [27]. In one of the biggest study including HDp the highest risk of death was in the group with SBP < 120 mmHg [28]. On the other hand, the lowest mortality rate was achieved with SBP higher than 160 mmHg. Also, in another study, which involved 37069 HDp, the highest risk of death was associated with SBP < 115 mmHg [29]. Also, in different studies, the results were very similar to above-cited article and showed that SBP > 135 mmHg in groups of HDp have a better outcome [30,31]. 

In our study in 2006, the predialysis BP was < 140/90 mmHg in 64.3% of HDp, but in 2016 this level was only achieved in 49.4% of HDp which may be related to older age and longer duration of dialysis treatment. The postdialysis goal that was < 130/80 mmHg was reached in 57.1% of HDp in 2006 and only in 27.1% in 2016. 

On the first look achieved values are not optimistic. However, they are very similar to results achieved by patients from the same unit with CKD in stage 1–4 [32] and better than values obtained by the general population in NATPOL 2011 study analyzing treatment of hypertension in Poland [33]. 

### 4.3. Limitations of the Study

There are some limitations of the study. First, the use of office BP readings to monitor the quality of BP control may be subject to significant error due to the white-coat syndrome and stress during hemodialysis. In reality, treatment results may be even better. On the other hand, the assessment of antihypertensive treatment effectiveness was based on a retrospective analysis of causal BP measurements, frequently following the morning dose of medications, which can significantly reduce the mean value of BP. Second, the surveys, in fact, were not performed on the same patients, and therefore, there is the possibility that differences in demographic and clinical characteristics between patients enrolled in particular surveys influenced the results. Also, we did not measure RRF and do not check side effects of diuretics. The exact doses of drugs and period of usage also were not measured. 

## 5. Conclusions

In summary, the results showed that during a ten-year period, the control of BP was worse. The main hypertensive treatment regimen is based on BB and CCB agents. Surprisingly, the usage of RAAS blockade and CCB decrease BP significantly. Additional studies concerning the control of BP and the role of RAAS blockade in HDp are necessary.

## Figures and Tables

**Table 1:** Characteristic of the study population.

Characteristic	2006	2011	2016	*p*
Number of participants (*n*)	56	81	85	--
Man (*n*/%)	35 (62.5)	49 (60.5)	53 (62.4)	ns
Mean age (years)	61.4 ± 12.5	64.4 ± 13.7	66.2 ± 14.4	<0.05
Average BMI (kg/m^2^)	25.8 ± 5.3	26 ± 5.5	25.2 ± 6.2	ns
Cardiovascular disease (*n*/%)	40 (71.4)	64 (79)	56 (65.9)	ns
Diabetes (*n*/%)	22 (39.3)	33 (40.7)	29 (34.1)	ns
Kt/v	1.52 ± 0.29	1.61 ± 0.27	1.66 ± 0.29	<0.05
Kt/v ≥ 1.4 (*n*/%)	41 (73.2)	67 (82.7)	72 (84.7)	ns
Duration of dialysis treatment (months)	23.4 ± 26.7	33.7 ± 25.4	45.1 ± 37.9	<0.05
Time of dialysis—median (minutes/week)	720	720	720	ns
Vascular access:				
Fistula (*n*/%)	49 (87.5)	68 (84)	70 (82.4)	ns
Catheter (*n*/%)	7 (12.5)	13 (16)	15 (17.6)	ns
Patients receiving EPO (*n*/%)	56 (100)	69 (85.2)	72 (84.7)	ns
Average dose of EPO (Unit)	4257	5026	5162	ns

**Table 2:** The value of blood pressure in hemodialysis patients.

Blood Pressure	2006	2011	2016	*p*
Average SBP predialysis (mmHg)	134.1 ± 17.5	130.4 ± 17.6	140.38 ± 20.3	<0.05
Average SBP postdialysis (mmHg)	123.7 ± 16.3	126.1 ± 18.0	138.9 ± 21.5	<0.05
Average DBP predialysis (mmHg)	77.09 ± 10.7	74.2 ± 8.8	76.13 ± 12.7	ns
Average DBP postdialysis (mmHg)	73.62 ± 8.6	72.5 ± 8.2	76.54 ± 11.6	ns
Blood pressure < 140/90 before HD (*n*/%)	36 (64.3)	52 (64.2)	42 (49.4)	ns
Blood pressure < 130/80 after HD (*n*/%)	32 (57.1)	47 (58)	23 (27.1)	<0.05

**Table 3:** The number of antihypertensive drugs used in the study population.

Number of Drugs	2006	2011	2016	*p*
Average number of drugs	2.25	2.43	2.53	<0.05
0 drugs (*n*/%)	6 (10.7)	6 (7.4)	8 (9.4)	ns
1 drug (*n*/%)	13 (23.2)	12 (14.8)	12 (14.1)	ns
2 drugs (*n*/%)	11 (19.6)	28 (34.6)	19 (22.4)	ns
3 drugs (*n*/%)	16 (28.6)	20 (24.7)	26 (30.6)	ns
4 drugs (*n*/%)	7 (12.5)	9 (11.1)	15 (17.6)	ns
5 drugs (*n*/%)	3 (5.4)	4 (4.9)	4 (4.7)	ns
6 drugs (*n*/%)	0	1 (1.2)	0	--
7 drugs (*n*/%)	0	1 (1.2)	1 (1.2)	--

**Table 4:** The groups of antihypertensive drugs used in the study population.

Group of Drug	2006	2011	2016	*p*
Beta-blockers (*n*/%)	35 (62.5)	54 (66.7)	63 (74.1)	ns
Calcium channel blockers (*n*/%)	28 (50)	36 (44.4)	45 (52.9)	ns
Alpha-blockers (*n*/%)	7 (12.5)	22 (27.2)	17 (20)	ns
ACEI (*n*/%)	22 (39.3)	25 (30.9)	17 (20)	<0.05
ARBs (*n*/%)	0	4 (4.9)	10 (11.8)	<0.05
RAAS blockade (*n*/%)	22 (39.3)	28 (34.6)	27 (31.7)	ns
Diuretics (*n*/%)	28 (50)	51 (63)	59 (69.4)	<0.05
Other (*n*/%)	5 (8.9)	5 (6.2)	3 (3.5)	ns

**Table 5:** The influence of hypertensive drugs for the value of blood pressure in the study population (linear regression).

**Year**	**2006**															
	**SBP Predialysis**				**SBP Postdialysis**				**DBP Predialysis**				**DBP Postdialysis**			
	**Univariate Analysis**		**Multivariate Analysis**		**Univariate Analysis**		**Multivariate Analysis**		**Univariate Analysis**		**Multivariate Analysis**		**Univariate Analysis**		**Multivariate Analysis**	
	**Value (mmHg)**	***p***	**Value (mmHg)**	***p***	**Value (mmHg)**	***p***	**Value (mmHg)**	***p***	**Value (mmHg)**	***p***	**Value (mmHg)**	***p***	**Value (mmHg)**	***p***	**Value (mmHg)**	***p***
Beta blockers	−3.87	ns	−7.13	ns	−2.46	ns	−0.54	ns	−1.81	ns	−3.42	ns	−0.58	ns	−0.63	ns
Calcium channel blockers	−4.32	ns	−6.81	ns	−4.13	ns	−1.59	ns	−1.70	ns	−1.02	ns	−0.65	ns	0.37	ns
Diuretics	−1.21	ns	−1.54	ns	−1.70	ns	−1.46	ns	−3.33	ns	0.73	ns	−5.21	ns	−3.21	ns
RAAS blockade	−6.69	<0.05	−8.96	<0.05	−4.62	<0.05	−2.96	ns	−2.48	ns	−3.26	ns	−0.98	ns	−0.95	ns
Alfa blockade	−3.70	ns	−1.8	ns	−2.88	ns	−1.46	ns	−1.04	ns	−0.29	ns	0.39	ns	0.63	ns
**Year**	**2011**															
	**SBP Predialysis**				**SBP Postdialysis**				**DBP Predialysis**				**DBP Postdialysis**			
	**Univariate Analysis**		**Multivariate Analysis**		**Univariate Analysis**		**Multivariate Analysis**		**Univariate Analysis**		**Multivariate Analysis**		**Univariate Analysis**		**Multivariate Analysis**	
	**Value (mmHg)**	***p***	**Value (mmHg)**	***p***	**Value (mmHg)**	***p***	**Value (mmHg)**	***p***	**Value (mmHg)**	***p***	**Value (mmHg)**	***p***	**Value (mmHg)**	***p***	**Value (mmHg)**	***p***
Beta blockers	−2.36	ns	−2.75	ns	−3.51	ns	−2.85	ns	−2.80	ns	−3.23	ns	−1.07	ns	−0.96	ns
Calcium channel blockers	−8.65	<0.05	−8.91	<0.05	−7.31	<0.05	−6.84	<0.05	−3.33	ns	2.45	ns	−3.22	<0.05	−3.43	<0.05
Diuretics	−1.9	ns	−1.10	ns	−0.09	ns	−0.36	ns	−3.12	ns	−2.10	ns	−6.61	ns	−0.95	ns
RAAS blockade	−3.83	ns	−3.55	ns	−3.28	ns	−2.38	ns	−2.91	ns	−2.13	ns	−1.32	ns	−1.38	ns
Alfa blockade	−3.37	ns	−1.91	ns	−5.29	<0.05	−1.10	ns	−2.63	ns	−2.06	ns	−1.31	ns	−0.70	ns
**Year**	**2016**															
	**SBP Predialysis**				**SBP Postdialysis**				**DBP Predialysis**				**DBP Postdialysis**			
	**Univariate Analysis**		**Multivariate Analysis**		**Univariate Analysis**		**Multivariate Analysis**		**Univariate Analysis**		**Multivariate Analysis**		**Univariate Analysis**		**Multivariate Analysis**	
	**Value (mmHg)**	***p***	**Value (mmHg)**	***p***	**Value (mmHg)**	***p***	**Value (mmHg)**	***p***	**Value (mmHg)**	***p***	**Value (mmHg)**	***p***	**Value (mmHg)**	***p***	**Value (mmHg)**	***p***
Beta blockers	−2.58	ns	−1.05	ns	−5.29	ns	−3.37	ns	−0.22	ns	−0.26	ns	−0.05	ns	−0.80	ns
Calcium channel blockers	−6.61	<0.05	−5.52	<0.05	−8.26	<0.05	−7.53	<0.05	−0.50	ns	−0.15	ns	−1.42	ns	−1.19	ns
Diuretics	−0.92	ns	−1.02	ns	−1.73	ns	−1.44	ns	−0.62	ns	−0.82	ns	−0.86	ns	−1.09	ns
RAAS blockade	−5.34	<0.05	−3.98	ns	−2.33	ns	−0.63	ns	−1.71	ns	−1.39	ns	0.15	ns	−0.82	ns
Alfa blockade	−3.93	ns	−0.92	ns	−3.74	ns	−0.44	ns	−2.20	ns	−2.04	ns	−2.27	ns	−2.34	ns
